# Modulation of cytokines and transcription factors (T-Bet and GATA3) in CD4 enriched cervical cells of Chlamydia trachomatis infected fertile and infertile women upon stimulation with chlamydial inclusion membrane proteins B and C

**DOI:** 10.1186/1477-7827-7-84

**Published:** 2009-08-22

**Authors:** Rishein Gupta, Harsh Vardhan, Pragya Srivastava, Sudha Salhan, Aruna Mittal

**Affiliations:** 1 Institute of Pathology-ICMR, Safdarjang Hospital Campus, Post Box no. 4909, New Delhi-110 029, India; 2 Department of Gynaecology & Obstetrics, Safdarjung Hospital, New Delhi-110 029, India

## Abstract

**Background:**

Chlamydial Inclusion membrane proteins (Incs), are involved in biochemical interactions with host cells and infecting Chlamydiae. We have previously reported the role of two Chlamydia trachomatis (CT) Incs, namely IncB and IncC in generating host immunity in CT infected women. Emerging data shows involvement of Inc stimulated CD4 positive T cells in aiding host immunity in infected fertile and infertile women through the secretion of interferon gamma. However the lack of data on the intra-cytokine interplay to these Incs in infected cell milieu prompted us to investigate further.

**Methods:**

A total of 14 CT-positive fertile, 18 CT-positive infertile women and 25 uninfected controls were enrolled in this study. CD8 depleted, CD4 enriched cervical cells were isolated and upon stimulation with IncB and IncC, modulation of cytokines (Interleukin (IL)-1 Beta, IL-4, IL-5, IL-6, IL-10, Interferon-gamma, IL-12, IL-23, Tumor Necrosis Factor-alpha and Granulocyte macrophage colony-stimulating factor (GM-CSF) and T cell lineage regulating transcription factors T-Bet and GATA3 was determined by real-time reverse-transcriptase (RT)-PCR and ELISA.

**Results:**

Significant higher expression (P < 0.05) of Interferon-gamma, IL-12, IL-23 and GM-CSF were found in Inc-stimulated CD4 enriched cervical cells of CT-positive fertile women and contrastingly high IL-1 Beta, IL-4, IL-5, IL-6 and IL-10 levels were found in CT-positive infertile women. Positive correlation (P < 0.05) was found between Interferon-gamma and T-Bet levels in CT-positive fertile women and IL-4 mRNA and GATA3 levels in CT-positive infertile patients upon IncB and IncC stimulation.

**Conclusion:**

Overall our data shows that CT IncB and IncC are able to upregulate expression of cytokines, namely interferon-gamma, IL-12, IL-23 and GM-CSF in CT-positive fertile women while expression of IL-1 Beta, IL-4, IL-5, IL-6 and IL-10 were upregulated in CT-positive infertile women. Our study also suggests that Incs are able to modulate expression of T cell lineage determinants indicating their involvement in regulation of immune cells.

## Background

*Chlamydia trachomatis *(CT) is an obligate intracellular pathogen and is the leading cause of sexually transmitted diseases (STD) globally [1] Chlamydial infection of the lower genital tract ascends to the upper genital tract and results in serious consequences to reproductive health, such as infertility, ectopic pregnancy, and pelvic inflammatory disease (PID) [2]. In India, a high chlamydial prevalence rate has been reported among symptomatic women with or without fertility-related disorders [3-6].

CT infection and propagation within host genital epithelial cells depends on a 48–72 hour unique biphasic developmental cycle, in which metabolically inert, infectious elementary bodies (EBs) enter host cells, replicate by binary division and differentiate into large reticulate bodies (RBs) within a specialized vacuole called the inclusion [7]. Chlamydial inclusion avoids fusing with components of the lysosomal pathway, yet maintain the selective ability for acquiring resource-laden host vesicles from the exocytic pathway, multivesicular bodies and lipid droplets. A family of secreted proteins, termed Inclusion membrane proteins (Incs), localized to the inclusion membrane (IM), exert crucial roles in vesicle fusion events [8]. Incs interact with host cell components and contribute to inclusion maturation and chlamydial development [9]. IncA, IncB and IncC are proteins encoded by the first three genes of Inc family respectively and are produced early in the chlamydial life cycle [7]. Antibodies to IncA, IncB and IncB have been detected in sera of infected animals and humans [10-12]. Antigen specific MHC class I-restricted CD8^+ ^T cell responses have also been reported for membrane associated incs [13-15].

Acquired immunity to chlamydial infection involves humoral and cell-mediated immune responses in infected individuals [16,17]. It is also reported that B cells and CD4^+ ^T cells are involved in providing immunity to CT infection [18,19]. Further, adaptive immune protection to CT involves recruitment of CD4^+ ^or CD8^+ ^T cells at sites of infection in nonhuman primate models or by adoptive transfer in mice models [20-25]. Furthermore, CD8^+ ^T cells play a minor protective role compared to that of CD4^+ ^T cells in genital chlamydial infection [20,26]. Cytokines produced by T cell subsets in response to CT infection are known to influence each other through stimulatory or inhibitory pathways and finally determine the clinical course of infection by resulting in successful clearance of CT or associated pathology [27]. Studies done in our laboratory suggests that CT IncB and IncC generate differential humoral and cell mediated immune responses in CT infected fertile and infertile women [28]. It was further observed that *invitro *Inc stimulation of CD4 positive enriched T cells obtained from these patients resulted in high levels of interferon gamma suggesting a probable role of CD4 mediated T helper (T_H _1) immune protection. In this study we further investigated (i) expression of cytokines upon IncB and IncC stimulation of CD4 positive enriched T cells in CT positive fertile and infertile women using cytokine specific RT-PCR and ELISA and (ii) correlation of cytokines expression with T_H _cell differentiation factors, namely T-Bet and GATA3.

## Methods

### Study population

A total of 35 symptomatic patients (having complaints of cervical discharge, cervicitis and infertility), attending the Gynaecology Outpatient Department of Safdarjung Hospital, New Delhi, India from March 2008 to May 2008 were randomly enrolled for the study. Twenty five healthy age-matched controls attending the family planning department for birth control measures were also enrolled. The study received approval from the hospital's ethics review committee and prior informed written consent was obtained from each patient. Procedures followed for sample collection were in accordance with the ethical standards for human experimentation established by the Declaration of Helsinki of 1975 (revised in 1983). At recruitment, a detailed clinical questionnaire was administered to each patient for collecting information on reasons for referral, gynaecology history including menstruation, symptoms of genital and urinary tract infection, obstetric and medical histories. Patients with positive urine pregnancy test, recent antibiotic therapy and history of recently treated sexually transmitted infection and genital tuberculosis were excluded from the study.

Fertile women were those having their last child birth within the last 4 months to 1 year. Infertile women were identified as those, which lack recognized conception after 1.5 to 2 years of regular intercourse without the use of contraception. The infertile group included women with referred diagnostic laparoscopy [29].

Based on clinical history and diagnosis, the patients were categorized into three groups. Group I comprised of CT-uninfected healthy controls with no infertility problems, Group II comprised of CT-positive fertile women and Group III comprised of CT-positive infertile women.

### Collection of samples

The vulva was examined for lesions and the cervix for warts, ulcers, ectopy, erythma and discharge, if any. A cotton tipped swab (Hi Media, Mumbai, India) was introduced into the cervical canal through a Cusco's speculum and after cleaning the endocervix with a cotton swab, endocervical swabs were collected from patients for diagnosis of CT and other STD pathogens. For collection of cervical cells, a cytobrush was placed within the endocervical canal so that the cells from the endocervical region and the zone between the endocervical and ectocervical regions (transformation zone) could be obtained. The cytobrush was then transferred to a sterile centrifuge tube containing sterile phosphate-buffered saline (PBS) (pH 7.2) supplemented with 100 U penicillin/ml, 100 μg streptomycin/ml and 100 μg glutamine/ml. No samples were collected from patients with friable cervix and contact bleeding to ensure collection of cervical lymphocytes only. Samples were then stored at 4°C, transported to the laboratory and processed within 1 h.

### Laboratory diagnosis

Endocervical samples were tested for chlamydial positivity by PCR analysis using CT specific 200 base pair (bp) plasmid primers [30]. In addition, PCR detection of CT *incB *(CT 232) and *incC *(CT 233) genes in all endocervical samples was performed as mentioned earlier [31]. Diagnosis for other STD pathogens was done as described earlier [29]. In brief, *Neisseria gonorrhoeae*, *Mycoplasma hominis *and *Ureaplasma urealyticum *were detected by culture while for diagnosis of *Candida sp*, bacterial vaginosis and *Trichomonas vaginalis *microscopy was done on gram stained smears.

### Expression of CT IncB and IncC proteins

Recombinant clones containing full length gene sequences of *incB *and *incC *cloned into pGEX expression vectors (Amersham Pharmacia Biotech Inc., NJ, USA) were obtained as a kind gift from Dr. Guangming Zhong at Department of Microbiology and Immunology, University of Texas Health Science Center at San Antonio, USA. These clones were further propagated in Terrific Broth (Amresco, Ohio, USA) and production of glutathione S-transferase (GST) fusion proteins was performed as described elsewhere [32]. Upon purification, fusion proteins were checked for consistency on sodium dodecyl sulphate (SDS)-polyacrylamide gels stained with a Coomassie blue dye (Sigma-Aldrich) and frozen at -80°C to be used in further assays.

### Antibody assays

Cervical washes of patients and controls were assayed for immunoglobulin G (IgG) antibodies to CT surface components using a commercially available ELISA (Ridascreen, R-Biopharm AG, Darmstadt, Germany). Results were obtained as mean absorbance of duplicated samples at A_450_. An OD > 1.1 was considered positive.

CT IncB and IncC specific IgG_2 _titres in cervical washes were determined by ELISA in 96-well plates coated with 1 μg antigen/well and 100 μL of cervical washes from patients and controls as previously described [33]. Positive samples were defined as those yielding an absorbance (OD) value at least two standard deviations (SDs) above the mean value obtained from the panel of samples taken from the negative subjects.

CT IncB and IncC specific IgG in cervical washes was further determined by Western blot assay as described previously [28]. Patients who detected presence of a 38 kDa band for IncB and a 44.4 kDa band for IncC were considered positive for western blot assay.

### Purification and proliferation of CD4 positive T cells

Cervical cells were isolated and counted as described earlier [34]. In brief, the tube containing the cytobrush was vortexed before removing the brush and the cells were filtered through a sterile 70-mm nylon cell strainer (Becton Dickinson, San Diego, CA, USA) to make a homogeneous preparation of cells. The population of the cell suspension was pelleted down at 1,500 rpm for 10 min to yield cervical cells. The viability of cells was determined by a trypan blue exclusion assay on a haemocytometer. CD8^+^T cells were positively selected from cervical cells using CD8 MACS MicroBeads^® ^(Miltenyi Biotec, CA, USA) according to manufacturer's instructions. The magnetically labelled CD8^+ ^T cells were retained in the column while the unlabelled cells which passed through the column were collected. The purity of CD8 depleted, CD4^+ ^T cells (collected as flow through) was determined on a FACS Caliber (BD Biosciences) using a PE-conjugated anti-CD4 monoclonal antibody (Becton Dickinson, San Jose, USA) (See Additional file 1). This CD8-depleted cell fraction was used for further assays and will be termed as CD4^+ ^T cells.

The CD4^+ ^T cells were further gently pelleted and suspended in RPMI-1640 medium (Sigma-Aldrich) supplemented with 10% heat-inactivated human AB serum and cultured in triplicate (2×10^5 ^cells/well) in round-bottomed 96-well plates with or without stimulants in a total volume of 200 μl. Subsequently cultures were incubated in humidified 5% CO_2 _at 37°C for 12 h (for real-time RT-PCR) and 72 h (for cell proliferation assays and cytokine specific ELISA).

### Stimulants

CT was grown on confluent McCoy cell monolayers at a multiplicity of infection 2 (ratio of number of infection forming units to the number of seeding cells was 2). Cells were lysed and whole EBs (CT positive control) were purified as described previously [35]. The concentration of CT Incs and other antigens was measured using Bradford assay (Sigma-Aldrich, MO, USA) according to manufacturer's instructions. IncB (1 μg/ml) and IncC (1 μg/ml) were used for all experiments. Phytohaemaglutinin (PHA 2 μg/ml) (Sigma-Aldrich) and free GST were used as positive control mitogen and negative control respectively in each experiment. Optimum concentrations of antigens and mitogen were determined in preliminary experiments as minimum concentrations giving maximal proliferation at different time intervals post stimulation.

### RNA extraction and real-time RT-PCR analysis for cytokines and transcription factors

Total RNA from IncB or IncC-stimulated CD4^+ ^T cells was isolated using RNeasyMini Kit (Qiagen, CA, USA), in accordance with the manufacturer's instructions and stored at -70°C. Complementary DNA (cDNA) was prepared using a SuperScript™ First-Strand Reverse Transcriptase kit (Invitrogen), in accordance with the manufacturer's instructions. The cDNA solution was diluted to 150 μl and stored at -20°C. All samples were reverse transcribed in a single batch and were all analysed for a given primer set in the same PCR run. The PCR amplification employed reagents supplied in a DyNAmo™ SYBR^® ^Green qPCR Kit (Finnzymes, Espoo, Finland), and each reaction volume (20 μl total) contained 5 μl of cDNA, and 0.5 μM of both primers. Sequences for endogenous control (β-actin) and cytokine genes (IL-1β, IL-4, IL-5, IL-6, IL-10, IL-12, TNF-α, IFN-γ and GM-CSF) and transcription factors (T-Bet and GATA3) used in this study mentioned in Table 1 were same as earlier reported by Jasper *et al*.[36]. All primers were of HPLC-purified grade and were commercially synthesized (MWG-Biotech AG, Ebersberg, Germany). The negative control included in each reaction consisted of H_2_O substituted for cDNA. PCR amplification was performed in an Applied Biosystems 7000 Real-Time PCR System (Applied Biosystems, CA, USA) under universal cycling parameters for relative quantification of cytokine expression in target samples according to the manufacturer's instructions (Applied Biosystems User Bulletin #2: Relative Quantitation of Gene Expression). For data analysis, the 2^-ΔΔCt ^method was used to calculate fold change [37].

**Table 1:** Details of primers used in this study

**No.**	**Cytokine gene primers**	**5'-3' sequence**
1	GM-CSF F*	AGC CCT GGG AGC ATG TGA
	GM-CSF R**	GAG TAG AGA CAC TGC TGC TGA GAT G
2	IL-β1F	GCT GAT GGC CCT AAA CAG ATG
	IL-β1R	ACGAAT CTC CGA CCA CCA CTA
3	IFNγ F	GAA ACG AGA TGA CTT CGA AAA GCT
	IFNγ R	ATG TCC AAC GCA AAG CAA TAC A
4	IL-12 F	TCG CGT TCA CAA GCT CAA GT
	IL-12 R	CA AAC CTG ACC CAC CCA AGA
5	TNF-α F	GCC CGA CTA TCT CGA CTT TGC
	TNF-α R	A ACC TTC CCA AAC GCC TCC
6	IL4 F	CCA CGG ACA CAA GTG CGA TAT
	IL4 R	CGT AAC AGA CAT CTT TGC TGC C
7	IL5 F	AAA GGC AAA CGC AGA ACG TT
	IL5 R	CTC TTG GAG CTG CCT ACG TGT
8	IL6 F	ACT CAC CTC TTC AGA ACG
	IL6 R	AGT AGT GAG GAA CAA GCC
9	IL10 F	GGG AGA ACC TGA AGA CCC TCA
	IL10 R	AAC AAG AGC AAG GCC GTG G
10	T-Bet(TBX21) F	AAC ACA GGA GCG CAC TGG AT
	T-Bet(TBX21) R	ATT GTG CTC CAG TCC CTC CA
11	GATA3 F	AGA TGG CAC GGG ACA CTA CCT
	GATA3 R	ATT AAG CCC AAG CGA AGG C
12	B-actin F	TGT GAT GGT GGG TAT GGG TC
	B-actin R	TAC AAT GAG CTG CGT GT

* Forward primer ** Reverse primer

### Quantification of secreted cytokines

Quantification of IL-1β, IL-4, IL-5, IL-6, IL-10, IL-12, IFN-γ, TNF-α, GM-CSF and IL-23 in culture supernatants of IncB or IncC-stimulated CD4^+ ^T cells was performed by commercially available ELISA kits (eBiosciences, San Diego, USA), in accordance with the manufacturer's instructions. The minimum detectable cytokine concentrations for these assays as provided by the manufacturer, were-IL-1β (4 pg/ml), IL-4 (2 pg/ml), IL-5 (4 pg/ml), IL-6 (2 pg/ml), IL-10 (2 pg/ml), IL-12 (4 pg/ml), IFN-γ (4 pg/ml), TNF-α (4 pg/ml), GM-CSF (2.5 pg/ml) and IL-23 (15 pg/ml).

### Statistical analysis

The Kruskal-Wallis non parametric test was used to compare continuous variables among multiple groups. The Mann-Whitney U test was used for comparing two groups. Categorical variables were compared using χ^2 ^test. The results were presented with 95% confidence interval (CI) and P < 0.05 was considered significant. All statistical analyses were performed with Graphpad Prism Version 5 (La Jolla, CA, USA).

## Results

### Study population

#### Laboratory diagnosis of CT

Cervical CT infection was diagnosed by CT specific 200 bp plasmid PCR in 35 patients. Three of these patients were found to be co-infected either with *Candida sp*., bacterial vaginosis, *T. vaginalis*, *M. hominis*, *U. urealyticum *or *N. gonorrhoeae *in the cervix and were thus excluded from the study. Based on clinical history and diagnosis, the patients were categorized into three groups. Group I (GI, n = 25) comprised of CT-uninfected healthy controls with no infertility problems, Group II (GII, n = 14) comprised of CT-positive fertile women and Group III (GIII, n = 18) comprised of CT-positive infertile women. The median ages of CT-positive infertile or fertile women and controls were comparable (28, 26 and 27 years, respectively).

#### Detection of CT incB and incC

Detection of *incB *and *incC *in cervical samples was done in all patients in group II (n = 14) and group III (n = 18) which were positive for CT specific 200 bp plasmid PCR. Using *incB *and *incC *PCRs, we detected positivity in group II (86%, 86%) and group III (94%, 100%) respectively (Table 2). Further, ELISA results showed that IgG_2 _antibodies to both IncB and IncC were significantly more prevalent in cervical washes of CT positive fertile and infertile patients compared to controls (P < 0.05) (Table 2). Western blot assay showed significantly higher positivity in cervical washes of CT positive fertile and infertile patients compared to controls (P < 0.05). Significantly higher number of women positively detected IncB and IncC IgG in CT positive fertile women as compared to CT-positive infertile women (Table 2).

**Table 2:** Detection of *C. trachomatis *positivity in study population by PCR, ELISA and Western blot

	ELISA			PCR		Western blot	
	CT IgG	IncB IgG_2_	IncC IgG_2_	IncB	IncC	IncB IgG	IncC IgG
GI (n = 25)	1 (4)	1(4)	2 (8)	0 (0)	0 (0)	1(4)	1(4)
GII (n = 14)	12 (86)^a, m^	10 (72)^b, m^	11 (79)^c, m^	12 (86)^d, m^	12 (86)^e, m^	9 (64)^f, m^	11 (79)^g, m^
GIII (n = 18)	9 (50)^m^	8 (44)^m^	7 (39)^m^	17 (94)^m^	18 (100)^m^	8 (44)^m^	7 (39)^m^

Note Table shows the number of patients who were positive for tests mentioned. Values in parenthesis denote percentage unless otherwise stated.

^a ^P = 0.029 as compared to GIII; ^b ^P = 0.010 as compared to GIII; ^c ^P = 0.007 as compared to GIII;^d ^P = NS as compared to GIII; ^e ^P = NS as compared to GIII; ^f ^P = 0.007 as compared to GIII, ^g ^P = 0.006 as compared to GIII; ^m ^P < 0.05 as compared to corresponding GI where,

Group I (GI) comprised of healthy women with no CT infection, Group II (GII) comprised of CT-positive fertile women, Group III (GIII) comprised of CT-positive infertile women. All categorical variables were compared using the χ^2 ^test.

### Cytokine mRNA expression levels in stimulated CD4^+ ^T cells

**Figure 1:**
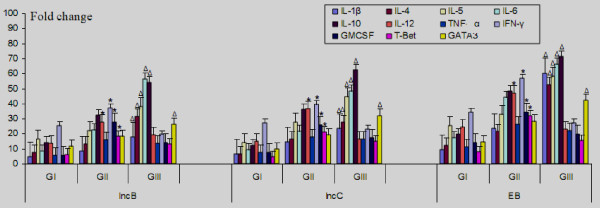
**mRNA expression of IL-1β, IL-4, IL-5, IL-6, IL-10, IL-12, TNF-α, IFN-γ, GM-CSF, T-Bet and GATA3 in CD4^+ ^cervical cells in GI, GII and GIII after *in vitro *stimulation with IncB, IncC and CT EB**. Real-time RT-PCR analysis of mRNA levels was done at 12 hours post infection where, Group I (GI) comprised of healthy women with no CT infection, Group II (GII) comprised of CT-positive fertile women, Group III (GIII) comprised of CT-positive infertile women. * P < 0.05 Expression of cytokine mRNA in GII compared to corresponding levels in GI and GIII by Kruskal Wallis test. Δ P < 0.05 Expression of cytokine mRNA in GIII compared to corresponding levels in GI and GII by Kruskal Wallis test. All cytokines were normalised against corresponding levels of β-actin endogenous gene. The graph show results as fold change represented by bars. Bars represent mean ± s.e.m. for all experiments. X axis- Stimulants used in the study; Y-axis- Fold change in RNA expression under different conditions.

**Figure 2:**
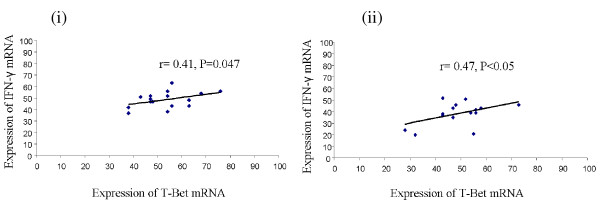
**Scatter plot showing positive correlation between IFN-γ and T-Bet in (i) IncB and (ii) IncC stimulated cells from CT-positive fertile women**.

**Figure 3:**
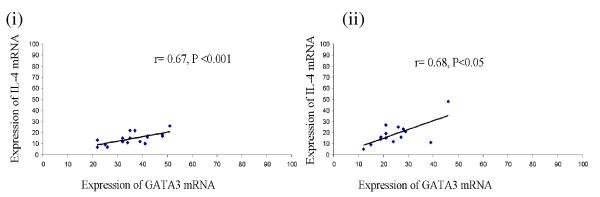
**Scatter plot showing positive correlation between IL-4 and GATA3 in (i) IncB and (ii) IncC stimulated cells from CT-positive infertile women**.

mRNA for cytokines viz. IL-1β, IL-4, IL-5, IL-6, IL-10, IL-12, TNF-α, IFN-γ and GM-CSF and transcription factors, T-Bet and GATA3 was detected in stimulated CD4^+ ^T cells in all patients' groups. On stimulation of CD4^+ ^T cells with IncB or IncC, significant increase in mRNA expression levels of IFN-γ, IL-12, GM-CSF (P < 0.05) was observed in cells obtained from CT-positive fertile women compared to controls and CT-positive infertile women. In contrast, IL-1β, IL-4, IL-5, IL-6, IL-10 mRNA expression levels were significantly higher (P < 0.05) in cells obtained from CT-positive infertile women compared to controls and CT-positive fertile women (Figure 1). On stimulation of CD4^+ ^T cells with IncB or IncC, IFN-γ and T-Bet levels were found to be positively correlated in CT-positive fertile women (Figure 2) whereas IL-4 mRNA levels were positively correlated to GATA3 expression in CT-positive infertile women (Figure 3).

### ELISA for cytokines in cell supernatants of stimulated CD4^+ ^T cells

Significantly higher levels of IL-1β, IL-6 and IL-10 were secreted from IncB or IncC stimulated CD4^+ ^T cells obtained from CT-positive infertile women as compared to CT-positive fertile women or controls (P < 0.05). In contrast, IncB and IncC stimulated CD4^+ ^T cells obtained from CT-positive fertile women secreted significantly higher levels of IL-12, IFN-γ, GM-CSF and IL-23 compared to CT-positive infertile women or controls (P < 0.05). Significantly high levels of TNF-α and IL-6 levels were secreted in CD4^+ ^T cells from CT-positive fertile and infertile women compared to controls (Table 3). IL-4 and IL-5 were below detection limit in all culture supernatants (data not shown). CD4^+ ^T cells stimulated with free GST showed no significant differences in levels of secreted cytokines (data not shown).

**Table 3:** Cytokine concentrations (pg/ml) in culture supernatants of stimulated cervical CD4^+ ^T cells

	**GI**			**GII**			**GIII**		
	**IncB**	**IncC**	**EBs**	**IncB**	**IncC**	**EBs**	**IncB**	**IncC**	**EBs**
**IL-1β**	**141.8** (109.5–335.5)	**169.3** (98.8–368.5)	**243.6** (145.1–467.9)	**312.3 **(109.8–489.2)	**407.1** (135.2–444.2)	**538.3** (124.8–767.3)	**692.9^a^** (167–941.4)	**825.3^b^** (182.8–982.3)	**900^c^** (288.6–1231.3)
**IL-6**	**146.7** (74.9–400.3)	**159.4** (87.3–548.5)	**299.92** (98.67–981.32)	**604^d^** (132.2–871.6)	**612.87^e^** (156.5–716.9)	**751^f^** (187.97–1093.2)	**900.63^a^** (441.4–1054.72)	**878.41^b^** (422.9–1078.78)	**1241.07^c^** (656.4–1878.3)
**IL-10**	**278.4** (132.7–254.2)	**287.9** (143.66–267.5)	**313.7** (159.4–623.9)	**500.3** (267.9–702)	**587.1** (249.3–734.2)	**643.9** (338.2–742.6)	**1132.1^a^** (562.6–1441.9)	**1278.3^b^** (600.3–1502.4)	**1538.4^c^** (768.2–1709.07)
**IL-12**	**187.6** (112.6–336.4)	**193.6** (123.2–414.1)	**265.9** (132.7–451.1)	**345.9^g^** (231.4–552)	**365.2^h^** (256.3–462.2)	**463.2^i^** (259.7–606.3)	**200.2** (168.4–378.4)	**189.7** (179.3–334.8)	**243** (182.1–356.2)
**IFN-γ**	**443.6^j^** (125.1–600.8)	**467.28^k^** (141–580)	**580.3^l^** (154.2–671.4)	**703.1^g^** (332.6–988.61)	**571.3^h^** (311.9–814.3)	**859.2^i^** (342.1–1460.5)	**229.5** (110.1–300.8)	**285.4** (154.2–412.6)	**453.3** (189.5–432.9)
**TNF-α**	**156.1** (72.5–254.3)	**167.3** (73.2–269.1)	**199.3** (82.2–343.5)	**298.3^d^** (138.2–400)	**271.9^e^** (128.3–438.6)	**486.1^f^** (188.2–569.2)	**248.6** (132.6–378.1)	**263.2** (128.97–350.6)	**411.2** (164.5–588.6)
**GM-CSF**	**181.2** (85.8–243.7)	**117.6** (93.6–229.7)	**233.1** (106.4–259.9)	**278.2^g^** (127.5–333.6)	**282.5^h^** (148.3–313.4)	**356.2^i^** (172.3–421.6)	**172.4** (99.8–223.8)	**186.7** (103.7–245.7)	**228.9** (141.6–279.6)
**IL-23**	**59.3** (43–176.5)	**76.2** (57.2–217.4)	**126.7** (105.6–211.3)	**153.2^g^** (65.7–200.1)	**134.7^h^** (87.2–213.2)	**187.4^i^** (148.2–231)	**75.2** (74.1–211)	**88.6** (68.9–202.6)	**147.5** (118.9–156)

Note. Data represents median values (bold). Values in parentheses depict range. All values in pg/ml unless otherwise stated. Along the horizontal axis are the stimulants IncB, IncC and EBs used in cells obtained from GI, GII and GIII and vertical axis are the cytokines secreted in cell cultures assayed using specific ELISAs.

^a, b, c ^P < 0.05 Level of secreted cytokine in GIII compared to respective levels in groups I and II by Mann Whitney U test.

^d, e, f ^P < 0.01 Level of secreted cytokine in GII compared to respective levels in group I by Mann Whitney U test.

^g, h, i ^P < 0.05 Level of secreted cytokine in GII compared to respective levels in groups I and III by Mann Whitney U test.

^j, k, l ^P < 0.05 Level of secreted cytokine in GI compared to respected levels in group III by Mann Whitney U test.

## Discussion

There is accumulating evidence on the role of CD4+ T cells in providing vigorous protective immunity aiding clearance of chlamydiae from host cells [21]. In this study, for detection of CT infection in patients, we have used 200 bp plasmid primers. Further the positivity for IncB and IncC was evaluated in CT positive patients only. However, since we have evaluated the IncB and IncC primers in limited CT positive samples, further study on large sample size is required to assess whether these primers can be used for detection of chlamydia infection. Antibodies to IncB and IncC in cervical washes were significantly higher in CT-positive fertile women compared to CT-positive infertile women. These results were similar and statistically significant to a commercial kit used for detection of antibodies to CT surface components. Antibodies are produced and are present in the genital tract secretions following CT infection, and the immunoprotective role of antibody-mediated immunity has also been reported [12,18]. CT IncA, B and C have been reported to be identified by their reactivity to convalescent sera from experimentally infected guinea pigs [10]. A major role for antibodies in chlamydial immunity is the enhancement of Th1 cell activation via Fc receptor (FcR)-mediated processes involving DCs. Moore *et al*. have reported that *in vitro *anti chlamydial antibodies increase the rate of Th1 cell activation by FcR^+/+ ^but not FcR^-/-^antigen-presenting cells, providing a mechanistic basis for need of both T-cell and humoral immune responses in protective immunity to chlamydial reinfection [38,39].

### Modulation of cytokines upon Inc-stimulation in CD4^+ ^cervical cells from CT-positive infertile women

Major histocompatibility complex (MHC)-class II-restricted CD4^+ ^T cells play a central role both by responding to, or by orchestrating, the activation of other immune components including cytokine production. In this study we detected high levels of IL-1β, IL-6, IL-4, IL-5 and IL-10 in IncB or IncC-stimulated CD4^+ ^T cells in CT-positive infertile women compared to CT-positive fertile women and controls. It has been reported that co-culture of CT serovar L2 with human blood monocytes induced the production of IL-1, a mediator of inflammation, tissue remodelling, and scarring [40]. Further, it has been reported that high levels of IL-1 have a toxic effect and cause severe tissue damage in a human Fallopian tube organ culture model following CT serovar D infection [41]. High levels of IL-1β and TNF-α have also been reported to be released from *in vitro *explant model upon infection with CT serovar E [42,43]. These cytokines are known to stimulate production of matrix metalloproteinases leading to tubal scarring during CT infection [44,45]. In addition to IL-1, TNF-α, alone or along with IFN-γ and GM-CSF are known to be involved in inducing IL-6 [46]. Although pleiotropic in nature, IL-6 is strongly correlated with pathogenesis in chlamydial infection. Persistent immune activation in the fallopian tube and elevated IL-6 levels due to CT infection has been reported [47]. CD4^+ ^T cells are considered the principle producers of IL-4 in an immune response [48], although other cell types, like mast cells, basophils, and CD4^- ^T cells, can produce IL-4 as well [49,50]. IL-4 itself has been shown to be a dominant factor for the induction of IL-4 expression in resting T cells. Further, expression of IL-4 can be induced in Th cells independent of IL-4 from non-Th cells [51,52]. In young sexually active adolescent women diagnosed with co-infection of oncogenic human papillomavirus and CT, IL-4 transcripts correlating to IL-10 have been detected in cervical samples [53]. Elevated levels of IL-4 and IL-5 in splenic lymphocytes from IFN-γ^--/-- ^mice following chlamydia-specific challenge and the inability in controlling local chlamydial infection and associated tissue damage and cellular infiltration has also been reported [54]. IL-10 is known to limit inflammatory and fibrotic damage and might enhance chlamydial persistence [55]. In the genital tract, elevated levels of IL-10 have been found in infertile women with documented CT infections [29,56]. Kinjyo *et al*. have characterized IL-10 as a Th3/Tr1 regulatory cytokine, showing the over production of IL-10 and TGF-β in the absence of a Th2 polarizing gene, SOC3, the suppressor of cytokine signalling [57]. Faal *et al*., found elevated levels of a T cell regulatory gene, forkhead box 3 (FOXP3), during active trachoma [58]. Additionally, Mark *et al*., had demonstrated diminished FOXP3 and IL-10 levels with early clearing of CT infection in a murine model [59]. The antagonistic role of IL-10 towards production of IFN-γ is also known. Yang *et al*., have reported that excessive IL-10 production in BALB/c mice inhibits Th1-like responses, including IFN-γ expression and the delayed-type hypersensitivity response following chlamydial infection, and consequently delays resolution of the infection [60]. Overall, these results indicate that IncB and IncC might be involved in pathogenesis caused due to CT infection in infertile women.

### Modulation of cytokines upon Inc-stimulation in CD4^+ ^cervical cells from CT-positive fertile women

We detected high levels of IL-12 and IL-23 in IncB or IncC-stimulated CD4^+ ^T cells from CT-positive fertile women compared to CT-positive infertile women and controls. IL-12, which is derived from monocytes and dendritic cells (DCs) is known to be important for the initial clearance of bacteria [61-64] and for promoting IFN-γ production by natural killer cells [65]. It has also been previously documented that clearance of chlamydial infection from female adolescents has been associated with decrease in IL-12 concentrations in endocervical samples [66]. We have previously reported increased levels of IL-12 in cervical secretions of CT-positive infertile women compared to cells in upper genital tract [29]. Further in another report, a high percentage of plasmocytoid DCs in cervical secretions of CT-positive infertile women [67] has suggested a possible mechanism recognition of CD4 or CD8 antigens on antigen presenting cells such as DCs. Phagocytosis of chlamydiae induces DCs to produce IL-12, which in turn promotes Th1 response and induces the presentation of chlamydial antigens to CD4^+ ^T-cells [68]. IL-23 is known to activate macrophages to produce pro-inflammatory cytokines and to improve antigen presentation by DCs [69]. In addition, IL-23 has been described as acting indirectly by inducing the production of Th1 polarizing cytokines, such as IL-12 and IFN-γ in murine DCs [70]. It has been reported that 60-kDa heat shock protein of *C. pneumoniae *promotes a Th1 immune response through IL-12/IL-23 production in monocyte-derived dendritic cells [71]. Thus, IL-23 fosters the ability of IL-12 to polarize the T cell response towards a Th1 pattern, which is protective to intracellular pathogens, but can be detrimental if the infection is not eradicated or if a re-infection occurs, leading to particularly strong activation of a Th1 response. Further, it has also been reported that IL-23, plays a central role in mediating chronic inflammatory responses and autoimmunity in mice [72,73]. IL-23 is also a regulatory factor that promotes IL-17-producing cells belonging to a discrete T cell subset termed as Th17 and distinct from the classical Th1 and Th2 cells [74,75]. Zhang *et al*., have recently reported the antichlamydial effect of MyD88-mediated IL-17 production which enhances inflammatory cytokine production and neutrophil infiltration during early stages of infection [76]. IL-17 is also known to significantly increase progesterone secretion by JEG-3 choriocarcinoma cells [77]. Contrastingly, it has been shown that CT serovar D infection in human trophoblast cells alters cellular cholesterol biosynthesis, depletes progesterone synthesis and impairs trophoblast implantation and placentation, thereby affecting pregnancy sequalae [78]. In this study, Azenabor *et al*., used whole chlamydial organisms and showed the mechanistic role of CT infection and the physiologic change meted on trophoblast. However, since the differential effects of chlamydial antigens on human reproduction is known [79], it is desirous to conduct further investigations to know the effects of Incs on host immunity through IL-17, a cytokine with significant roles in both immune-mediated pathologies and protection against microbial infections [80,81].

IncB or IncC-stimulated CD4^+ ^T cells from CT-positive fertile women produced IFN-γ and GM-CSF at levels higher than in CT-positive infertile women and controls. CD4^+ ^and CD8^+ ^T cells are reported to be sources of IFN-γ production in CT infection although the former is the major contributor [82]. Further, the antichlamydial activity of CD4^+ ^T cells is primarily associated with the production of high levels of IFN-γ [83-85]. IFN-γ reportedly promotes the destruction of CT [86] but also triggers macrophage release of inflammatory mediators that cause fibroblast proliferation, thereby enhancing the synthesis of collagen. In addition, IFN-γ delays the developmental cycle of CT so that chlamydial reticulate bodies persist longer, which might result in persistent unapparent infection and also, play a role in immunopathogenesis by promoting inflammatory damage and fibrosis [87]. However in spite of the dual functionality of IFN-γ, there is accumulated evidence to suggest that Th1 responses and IFN-γ production are important for optimal resolution of genital chlamydial infection [1,19]. GM-CSF is known to activate macrophages and up-regulate CD14 and MHC class II expression resulting in more efficient antigen presentation and development of protective immunity [88]. Further, in these cells, IncB or IncC-stimulation produced TNF-α higher than in controls. TNF-α along with IFN-α and IFN-γ has been shown to synergistically enhance inhibition of CT serovar D growth *in vitro *[89].

### Modulation of transcription factors, T-Bet and GATA3 upon Inc-stimulation

In this study we found elevated expression of transcription factor T-Bet in Inc- stimulated CD4^+ ^T cells from CT-positive fertile and infertile women compared to controls. Expression of T-Bet was found to be higher in cells of CT-positive fertile women compared to CT-positive infertile women. T-Bet has been identified as a Th1 cell-specific factor that induces the production of IFN-γ by developing Th2 cells. T-Bet is also reported to be involved in chromatin remodelling of the gene that encodes IFN-γ, induction of expression of the IL-12 receptor β2-subunit (IL-12Rβ2) and stabilizing its own expression, either through an intrinsic autocatalytic loop or the autocrine effects of IFN-γ signalling [90]. Although both CD4^+ ^and CD8^+ ^T cells, as well as NK cells express T-Bet, there is less dependence on T-Bet for high-level expression of IFN-γ in CD8^+ ^T cells than in CD4^+ ^T cells [91]. Further, the importance of T-Bet for the development of Th1 responses *in vivo *is highlighted by the susceptibility of T-Bet knock-out mice to challenge with *Leishmania major *and their predisposition to allergic airway disease [92]. Thus higher expression of T-Bet in CT-positive fertile women compared to CT-positive infertile women is indicative of differentiation of native Th0 cells towards a CD4^+^-Th1 mediated protective response for clearance of CT.

Elevated expression of GATA3 transcription factor was detected in Inc stimulated CD4^+ ^T cells from CT-positive infertile women compared to CT-positive fertile women and controls. GATA3 is a zinc-finger transcription factor and is crucial for inducing key attributes of Th2 cells including transcriptional competence of the Th2 cytokine cluster, which includes the genes encoding IL-13, IL-4 and IL-5 [93,94].

Overall, our data suggests that Th1 mediated immune responses in CD4^+ ^T cells can contribute to protection and clearance of genital chlamydial infection. However, Th2 mediated responses can be detrimental and contribute towards pathology of genital tracts and lead to long term reproductive sequalae of CT infection in infected hosts.

In this study we investigated the role of CT IncB and IncC specific immune responses in CD4^+ ^cervical cells from infected women by evaluating cytokine expression using quantitative real time PCR and ELISA. These two methods were selected as we were interested in studying expression of cytokines at transcription and secretory levels. IL-4 and IL-5 were detected only at the genomic level however their levels were below detection limits in culture supernatants by ELISA. It has been previously reported that the amount of IL-4 secreted is not proportional at mRNA level [95]. Further, the subcellular localization, translation and decay of cytokine transcripts and the overall post-transcriptional control is critical in determining the amount of IL-4 and IL-5 production in cells involved in immune responses [96,97]. Although TNF-α mRNA transcripts were detected, the levels in-between experimental groups were of physiologically significance only in culture supernatant secretions. It has been previously shown that TNF-α expression is controlled at transcriptional and translational levels and is the basis for discrepancy between RT-PCR and ELISA data [98,99]. Our study also indicates that RT-PCR can be used for a relatively low number of cells and could be efficient for simultaneous detection of multiple cytokines, especially those undergoing post-transcriptional changes [100,101].

This study reiterates the importance of T-helper (Th) responses in anti-chlamydial immunity with emphasis on the contribution of CD4^+ ^T cells. Our study is in contrast with previous reports on MHC Class I restricted CD8^+ ^T cell recognition of Inc antigens derived from the cytosol [13-15]. Nonetheless, as it has been well established that both MHC Class I and II restricted pathways are involved in presenting antigens to immunocompetent cells, the simultaneous activation of both pathways by Inc antigens can be explained. Incs localized to the IM are thought to play important roles in exchanging both materials and signals with host cells via the IM in order to establish and maintain a successful intravacuolar growth [102-104]. Further, as human chlamydial vaccine research efforts are underway to identify immunogenic proteins containing adequate epitopes to elicit a vigorous Th1 and a sufficient Th2 response, and antibody epitopes to induce a humoral immune response required for optimal protection from reinfection, the role of incs to this end are of interest.

## Competing interests

The authors declare that they have no competing interests.

## Authors' contributions

RG performed experiments mentioned in the manuscript. HV and PS assisted in Western blotting experiments. SS and PS helped in enrollment of patients for the study. AM and RG have analysed the data and written the manuscript. All authors have read and approved the final manuscript.

## Supplementary Material

Additional file 1 **Supplementary figure 1: Flowcytometric analysis of CD4 cervical T cell**. CD8 T cells were positively selected from cervical cells using CD8 MACS MicroBeads^®^. The purity of enriched CD4^+ ^T cells was determined using a PE-conjugated anti-CD4 monoclonal antibody. Click here for file
