# Supplementary material for: Modulation of cytokines and transcription factors (T-Bet and GATA3) in CD4 enriched cervical cells of Chlamydia trachomatis infected fertile and infertile women upon stimulation with chlamydial inclusion membrane proteins B and C
Source: Reprod Biol Endocrinol. 2009 Aug 22;7:84. doi: 10.1186/1477-7827-7-84 (PMC2736965; doi:10.1186/1477-7827-7-84)
Supplement: Additional file 1 — Supplementary figure 1: Flowcytometric analysis of CD4 cervical T cell. CD8 T cells were positively selected from cervical cells using CD8 MACS MicroBeads®. The purity of enriched CD4+ T cells was determined using a PE-conjugated anti-CD4 monoclonal antibody. [file 1477-7827-7-84-S1.doc]

Supplementary figure 1

Supplementary Fig.1. Flowcytometric analysis of CD4 cervical T cell

CD8 T cells were positively selected from cervical cells using CD8 MACS MicroBeads®. The purity of enriched CD4^+^ T cells was determined using a PE-conjugated anti-CD4 monoclonal antibody.
